# Supplementary material for: Transcriptome Analysis Reveals Vimentin-Induced Disruption of Cell–Cell Associations Augments Breast Cancer Cell Migration
Source: Cells. 2022 Dec 13;11(24):4035. doi: 10.3390/cells11244035 (PMC9776984; doi:10.3390/cells11244035)
Supplement: Supplementary file 1 [file cells-11-04035-s001.zip › DEGs and enrichment analysis.zip!/DEGs and enrichment analysis/WT_VIMvsVector GO enrichment/DOWN/WT_VIMvsVector_down.BPbar.pdf]

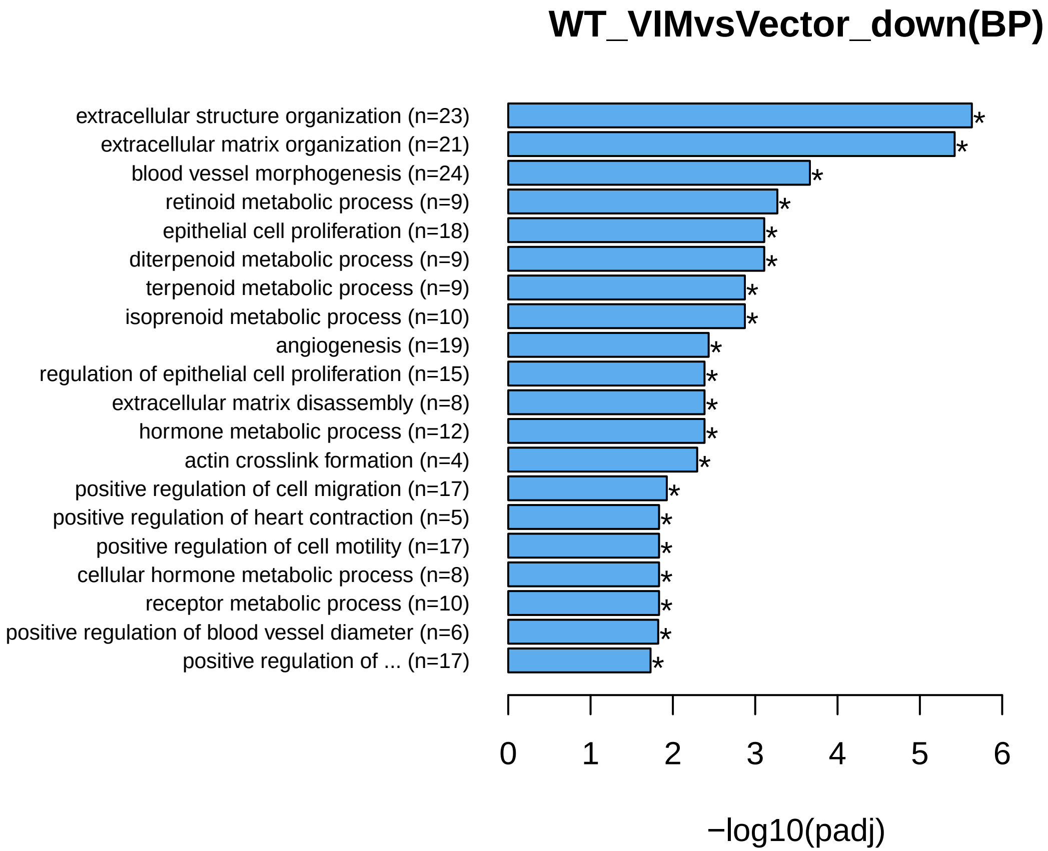
WT_VIMvsVector_down(BP)
extracellular structure organization (n=23)
extracellular matrix organization (n=21)
blood vessel morphogenesis (n=24)
retinoid metabolic process (n=9)
epithelial cell proliferation (n=18)
diterpenoid metabolic process (n=9)
terpenoid metabolic process (n=9)
isoprenoid metabolic process (n=10)
angiogenesis (n=19)
regulation of epithelial cell proliferation (n=15)
extracellular matrix disassembly (n=8)
hormone metabolic process (n=12)
actin crosslink formation (n=4)
positive regulation of cell migration (n=17)
positive regulation of heart contraction (n=5)
positive regulation of cell motility (n=17)
cellular hormone metabolic process (n=8)
receptor metabolic process (n=10)
positive regulation of blood vessel diameter (n=6)
positive regulation of ... (n=17)
0
1
2
3
4
5
6
−log10(padj)
